# Brain hemispheres with right temporal lobe damage swap dominance in early auditory processing of lexical tones

**DOI:** 10.3389/fnins.2022.909796

**Published:** 2022-08-26

**Authors:** Yarui Wei, Xiuyuan Liang, Xiaotao Guo, Xiaoxiao Wang, Yunyi Qi, Rizwan Ali, Ming Wu, Ruobing Qian, Ming Wang, Bensheng Qiu, Huawei Li, Xianming Fu, Lin Chen

**Affiliations:** ^1^Biomedical Engineering Center, School of Information Science and Technology, University of Science and Technology of China, Hefei, China; ^2^Department of Magnetic Resonance Imaging, The First Affiliated Hospital of Zhengzhou University, Zhengzhou, China; ^3^Department of Neurobiology and Biophysics, School of Life Sciences, University of Science and Technology of China, Hefei, China; ^4^Department of Otolaryngology-Head and Neck Surgery, The First Affiliated Hospital, University of Science and Technology of China, Hefei, China; ^5^Department of Rehabilitation Medicine, The First Affiliated Hospital, University of Science and Technology of China, Hefei, China; ^6^Department of Neurosurgery, The First Affiliated Hospital, University of Science and Technology of China, Hefei, China; ^7^Clinical Hearing Center, Affiliated Eye and ENT Hospital, Fudan University, Shanghai, China

**Keywords:** hemisphere dominance, lexical tone, mismatch negativity, stroke, brain lesion

## Abstract

Labor division of the two brain hemispheres refers to the dominant processing of input information on one side of the brain. At an early stage, or a preattentive stage, the right brain hemisphere is shown to dominate the auditory processing of tones, including lexical tones. However, little is known about the influence of brain damage on the labor division of the brain hemispheres for the auditory processing of linguistic tones. Here, we demonstrate swapped dominance of brain hemispheres at the preattentive stage of auditory processing of Chinese lexical tones after a stroke in the right temporal lobe (RTL). In this study, we frequently presented lexical tones to a group of patients with a stroke in the RTL and infrequently varied the tones to create an auditory contrast. The contrast evoked a mismatch negativity response, which indexes auditory processing at the preattentive stage. In the participants with a stroke in the RTL, the mismatch negativity response was lateralized to the left side, in contrast to the right lateralization pattern in the control participants. The swapped dominance of brain hemispheres indicates that the RTL is a core area for early-stage auditory tonal processing. Our study indicates the necessity of rehabilitating tonal processing functions for tonal language speakers who suffer an RTL injury.

## Introduction

The hemispheric specialization of language has been evaluated in multiple studies over the years, and the general consensus is that the left hemisphere is specialized for the processing of speech (Broca, 1861; Wernicke, 1874), whereas the right hemisphere is specialized for the processing of pitch such as musical tones (Zatorre et al., 2002; Poeppel, 2003). As to the factors responsible for this labor division between the two hemispheres, the functional hypothesis claims that the division depends on the auditory cues that serve as input signals (Whalen and Liberman, 1987; Liberman and Whalen, 2000), whereas the acoustic hypothesis claims that the division depends on the acoustic properties of input signals. Thus, the functional hypothesis predicts that linguistic pitch such as lexical tones is preferentially processed in the left hemisphere, whereas the acoustic hypothesis predicts that pitch is preferentially processed in the right hemisphere (Zatorre and Belin, 2001; Zatorre et al., 2002; Albouy et al., 2020). Moreover, a recent study reported increased activation in the right hemisphere when comprehending noisy spoken sentences in Mandarin Chinese (Song et al., 2020). As a matter of fact, neither of these two competing hypotheses can account for the full range of experimental data (Shankweiler and Studdert-Kennedy, 1967, 1975; Shtyrov et al., 2000).

Tonal languages such as Mandarin Chinese deploy lexical tones together with consonants and vowels to define word meaning. Previous neuroimaging studies, including positron emission tomography (PET) and functional magnetic resonance imaging (fMRI), have reported that the bilateral superior temporal gyri (STG), the left anterior insula cortex, and the left middle temporal gyrus, as well as the right lateralized cortical activations in the posterior inferior frontal gyrus, are activated during the processing of lexical tone in Mandarin Chinese (Klein et al., 2001; Wong et al., 2004; Liu et al., 2006; Xi et al., 2010; Chang et al., 2014). In our previous study, spectrograms of the syllable /bai/ pronounced in four lexical tones (bai1, bai2, bai3, and bai4) illustrated that the lexical tones are characterized by varying frequencies with time, and that lexical tones have minimal effects on the voice onset time of the consonant /b/; moreover, spectrograms of the syllables /bai, /dai, and /tai/ pronounced in a flat tone (bai1, dai1, and tai1) illustrated that the syllables show relatively unchanged frequencies with time and that the consonants in the upper syllables are characterized by temporal variations as reflected by the voice onset time. Therefore, lexical tones and consonants are ideal materials for testing the two hypotheses. In our previous study (Luo et al., 2006), we proposed that the processing of a lexical tone carrying semantic information is lateralized to the right hemisphere at an early stage, but to the left hemisphere at a late stage. This so-called two-stage model (Luo et al., 2006) claims that hemisphere labor division initially depends on the acoustic properties of input signals and then depends on the functional cues in the processing from sound to meaning. Thus, the acoustic hypothesis and the functional hypothesis are not mutually exclusive, with each representing a different temporal stage of processing (Ren et al., 2009; Zhou et al., 2021). Our two-stage model resolves the debate over the cues that are used by the brain for the processing of speech sound and tonal sound.

The role of the right hemisphere in speech comprehension (Gainotti et al., 1981; Posner and Petersen, 1989; Mitchell and Crow, 2005; Lam et al., 2016; Gajardo-Vidal et al., 2018) and the speech impairments in patients with right brain damage (Gandour et al., 1988; Hagoort et al., 1996; Mitchell and Crow, 2005; Kadyamusuma et al., 2011; Gajardo-Vidal et al., 2018) have been explored since the 1980s. However, issues related to brain labor division for linguistic processing became more complicated in clinical observations. Some studies have shown that patients with left brain damage show less left lateralization in early auditory processing of consonants (Becker and Reinvang, 2007) and impairments in tone tasks for tonal languages (Gandour et al., 1992, 1996). Other studies have shown impairments of tone identification and production (Kadyamusuma et al., 2011) as well as the acoustic pattern (Gandour et al., 1988) in patients with right brain lesions. Patients with brain lesions are ideal subjects for investigating these issues. However, previous studies were mostly conducted at a behavioral level, which reflects auditory processing at a late stage, not an early stage. Thus, the impairments in the processing of lexical tones at an early stage, or a preattentive stage, and the influence of injury on the labor division of the brain hemispheres for auditory tonal processing at the electrophysiological level remain unclear. We believe that the right hemisphere dominance in early auditory processing of lexical tones would be impaired at the electrophysiological level in patients with right brain lesions. Considering the critical role of the right temporal lobe (RTL) for lexical-tone processing (Ge et al., 2015; Si et al., 2017; Liang and Du, 2018), we predicted that the impairments would be apparent in patients with RTL lesions but not in those with right non-temporal lobe (RNTL) lesions.

In the present study, we explored the hemisphere dominance in early auditory processing of lexical tones by using whole-head electric recordings of mismatch negativity (MMN) obtained from native Mandarin Chinese-speaking patients with RTL or RNTL lesions under a passive auditory oddball paradigm (Picton et al., 2000). The MMN is an index of the brain's automatic processing at an early stage (Naatanen et al., 1978), and it has been used as a probe in several studies related to the realm of pitch and music, as well as language (Luo et al., 2006; Chobert et al., 2012; Wang et al., 2013). For the source localization of MMN, many neuroimaging studies such as PET, fMRI, and magneto/encephalography (M/EEG) have proposed that beyond the bilateral STG, the right inferior frontal gyrus (IFG) contributes to MMN generation (Rinne et al., 2000; Opitz et al., 2002; Dura-Bernal et al., 2012). For the purpose of comparison, we also measured the MMN evoked with pure tones with varied frequencies, which are non-speech stimuli and are known to be dominantly processed in the right brain (Schonwiesner et al., 2005). We used the shortened Mandarin Chinese version of the Token test (De Renzi and Faglioni, 1978) to measure whether right brain injury impairs the ability of speech comprehension for Mandarin Chinese.

## Materials and methods

Informed consent was obtained from the participants in accordance with the Declaration of Helsinki. The research protocols used in this study were approved by the Ethics Committee of the First Affiliated Hospital, University of Science and Technology of China.

### Participants

Patients participating in this study were recruited from the First Affiliated Hospital, University of Science and Technology of China and screened using the following criteria: (i) provided informed consent after the procedure had been fully explained; (ii) native speakers of Mandarin Chinese; (iii) lesions were restricted to the right hemisphere, i.e., RTL and RNTL; (iv) right-handed before stroke onset, and musically untrained; (v) no contraindications to magnetic resonance imaging (MRI); and (vi) no medical history of audiological, mental, or neurological problems before stroke. These criteria were met in 24 patients: 11 patients with stroke in the RTL (age, 37–76 years; mean age, 57 years; two females) and 13 patients with stroke in the RNTL (age, 37–63 years; mean age, 53 years; two females). Brain lesions in these 24 patients were caused by a stroke with cerebral infarction (10 RTL: 11 RNTL) or cerebral hemorrhage (one RTL: two RNTL). Demographic, clinical, and lesion data of each patient are shown in Supplementary Table 1. Fourteen healthy age- and sex-matched control participants (age, 42–64 years; mean age, 52 years; five females) with Mandarin Chinese as their native language also volunteered to participate in this study (Supplementary Table 1). These participants were not musically trained and did not have a medical history of audiological, mental, or neurological diseases. All participants were right-handed in the assessment performed with the Edinburgh Handedness Inventory (Oldfield, 1971). The hearing thresholds of the three groups were tested by pure-tone audiometry at 500, 1, 2, and 4 kHz, as in a previous study (Robson et al., 2014). We first performed a Shapiro–Wilk test to identify whether the hearing thresholds for each group were normally distributed, and the results showed that the hearing thresholds were not normally distributed for some parts of the groups. Then, we performed the Kruskal–Wallis *H*-test to determine whether the hearing thresholds among the groups were matched, and the results showed no significant difference among the groups (the left ear: $X_{\left(2\right)}^{2}$ = 5.40, *P* > 0.05; the right ear: $X_{\left(2\right)}^{2}$ = 3.63, *P* > 0.05). The data for the hearing thresholds of subject No. 8 in the RTL group and subject No. 3 in the RNTL group were not collected. The patients reported no hearing problems before stroke.

### Lesion overlay map

**Figure 1:**
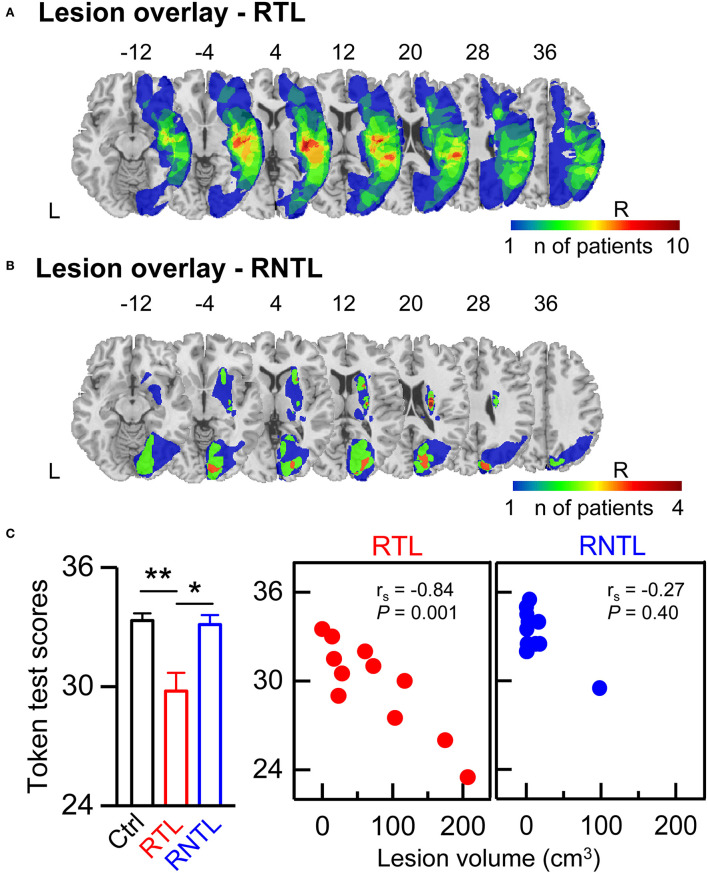
Lesion overlay map and speech comprehension ability. **(A,B)** The lesion overlap map of patients with right temporal lobe (RTL) damage **(A)** and right non-temporal lobe (RNTL) damage **(B)**. The heat map displays the number of patients with lesions in that respective area. Coordinates refer to MNI space. **(C)** Token test scores in each group. Token test scores of the RTL group were lower than that of the control group and that of the RNTL group (Left). The values are expressed as mean ± SE. *P < 0.05, **P < 0.01. Significantly negative correlation was found between lesion volume and Token test scores in the RTL group only (Central), but not the RNTL group (Right). *r*_s_ represents the correlation coefficient. L, left; R, right.

Structural high-resolution MRI scans of 13 patients (four RTL: nine RNTL) and CT scans of 11 patients (seven RTL: four RNTL) were acquired. The MRI scans were acquired on a 3T Philips Achieva scanner and included good T2-weighted or DWI B0 images. The CT scans were acquired on a Siemens scanner or a Philips scanner. Lesions were manually delineated by an experienced neurologist in the axial plane on each slice of the T2-weighted (eight RNTL patients; slice thickness, 5 or 5.5 mm; in-plane resolution, 1 mm), DWI (four RTL and one RNTL patients; slice thickness, 5 or 5.5 mm; in-plane resolution, 2 mm), or CT images (slice thickness, 5 mm; in-plane resolution, ≤ 0.5 mm) by using MRIcron (Rorden and Brett, 2000). Lesion volume was computed by multiplying the damaged area on each delineated slice by the slice thickness. The T2, DWI, and CT images of patients were transformed into standard stereotactic space (MNI) by using a clinical toolbox (www.nitrc.org/projects/clinicaltbx/) and SPM12 (www.fil.ion.ucl.ac.uk/spm). These images were resampled to yield the same voxel resolution, i.e., 1 mm^3^. The lesion overlay maps for the RTL (Figure 1A) and RNTL (Figure 1B) groups displayed a distributed profile at the group level. Inspection of the lesion overlay maps and individual MRI/CT scans indicated that the patients in the RTL group mainly had lesions in the RTL, the right insular, and the right frontal lobe (Supplementary Table 1 and Figure 1A), and those in the RNTL group mainly had lesions in the right periventricular white matter, the right basal ganglia, and the right occipital lobe (Supplementary Table 1 and Figure 1B).

### Stimuli

Lexical tones and pure tones were used as stimuli in this study. Lexical-tone stimuli were obtained and slightly modified from those used in our previous study (Luo et al., 2006), in which the Mandarin consonant-vowel (CV) syllables /bai1/ and /bai4/ were employed and originally pronounced by an adult male Mandarin speaker (Sinica Corpus, Institute of Linguistics, Chinese Academy of Social Sciences, Beijing, China). Pure tones were generated by Audition 3.0 (Adobe Systems Inc., Mountain View, CA, USA). The duration of each lexical tone was normalized to 350 ms, the duration of each pure tone was 200 ms, and both included a 5-ms linear rise and fall time. The lexical-tone contrast was created by a sequence of /bai1/ frequently presented as the standard stimuli and /bai4/ infrequently presented as the deviant stimuli during the auditory stream. The pure-tone contrast was created by a sequence of pure tones frequently presented at 550 Hz as the standard stimuli and infrequently presented at 350 Hz as the deviant stimuli.

### Procedure

Two blocks including the lexical-tone contrast and the pure-tone contrast were separately and randomly presented to the participant in one session with a 5-min break. Each block consisted of 800 trials. The participants were instructed to watch a silent movie and ignore the auditory stimuli they heard. The detection thresholds of lexical tones and pure tones were measured first, and all stimuli were then presented binaurally at 78 dB above the detection thresholds for each listener through headphones (TDH-39; Telephonics, Farmingdale, NY, United States) in an electrically shielded soundproof room. The standard stimuli were presented with a probability of 7/8 and the deviant stimuli were presented with a probability of 1/8. The stimulus order was pseudorandomized while maintaining a restriction that each deviant stimulus was separated by at least two standard stimuli. The inter-stimulus onset interval was 550 ms for the lexical-tone contrast and 500 ms for the pure-tone contrast.

### Data collection and analysis

The EEGs were recorded with 17 Ag/AgCl electrodes (Brain Products GmbH, Munich, Germany) placed at the standard electrode sites (F3, Fz, F4, FC1, FC2, FC5, FC6, C3, Cz, C4, P3, Pz, P4, FCz, Fpz, left mastoid, and right mastoid) according to the extended international 10–20 system. Two electrodes were used to measure the vertical and horizontal electrooculograms (EOGs). The reference electrode was attached at FCz and the ground electrode was placed between Fpz and Fz. Current signals (0.1–100 Hz) were continuously recorded by BrainAmp DC amplifier and sampled at 500 Hz. Impedances were maintained at <5 kΩ for all electrodes. The EEG and EOG data were recorded online and digitized using Brain Vision Recorder software (Brain Products, Munich, Germany).

Data from the head recordings were processed offline using Brain Vision Analyzer software (Brain Products, Munich, Germany). The recording was rejected when it was evidently contaminated by the EMG signal. An automatic ocular correction was then performed. Data were re-referenced to the average of the left and right mastoids and filtered (1–30 Hz). Epochs obtained from the continuous data were 600 ms in length, including a 100-ms pre-stimulus baseline, and were rejected when fluctuations in amplitude were >100 μV. The event-related potentials evoked by the standard and the deviant stimuli were calculated by averaging individual trials. MMN was derived from a different wave by subtracting the event-related potential evoked by the standard stimuli from that evoked by the deviant stimuli (Picton et al., 2000; Naatanen et al., 2004). Scalp topographic maps were produced using Brain Electric Source Analysis (MEGIS Software GmbH, Munich, Germany).

### Speech comprehension test

We measured the speech comprehension ability of Mandarin Chinese participants by using the shortened Mandarin Chinese version of the Token test (De Renzi and Faglioni, 1978). The materials consisted of tokens of different colors (white, blue, yellow, red, and green), shapes (squares and circles), and sizes (large and small). The examinee followed verbal instructions that increased in complexity from simple commands (e.g., “Touch a circle”; “Touch the red circle”) to more challenging commands such as “Before touching the yellow circle, pick up the red square.” The Token test scores were adjusted for years of education. Adjusted scores between 25 and 28 were regarded as an indicator of mild comprehension problems, those between 17 and 7 indicated moderate problems, and those below 17 indicated severe or very severe problems. The Token test scores of subject No. 3 in the RNTL group were not collected.

### Statistical analysis

**Figure 2:**
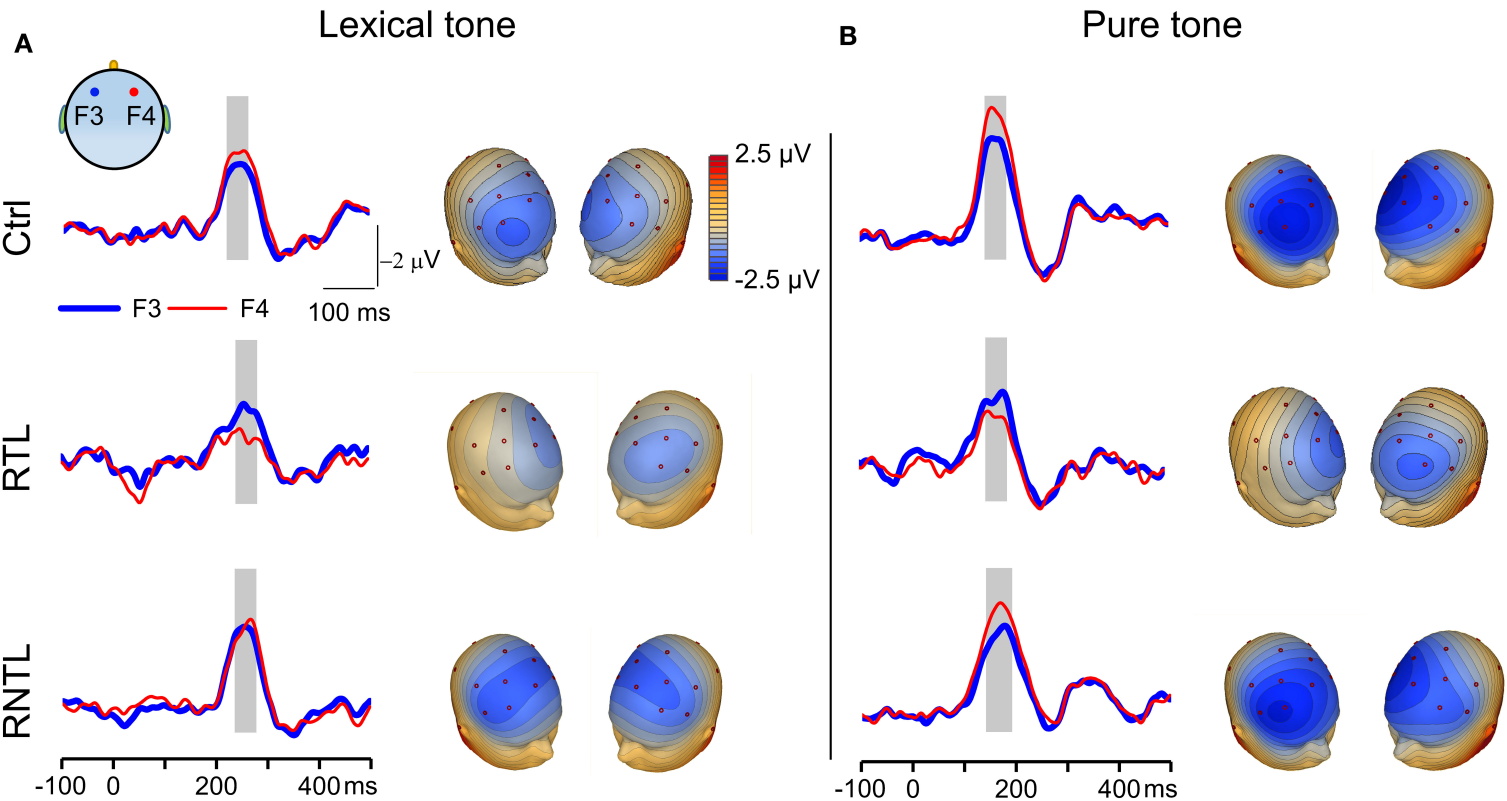
MMN responses recorded on the left and right sides of the scalp. **(A)** Grand average traces of MMN evoked by the lexical-tone contrast were recorded from one pair of electrodes on the left (F3, thick blue lines) and right (F4, thin red lines) sides in the control (Upper, *n* = 14), RTL (Central, *n* = 11), and RNTL (Lower, *n* = 13) groups (Left). Gray bars indicate the time window in which MMN amplitude was calculated. Scalp topographic maps constructed from grand average MMN evoked by the lexical-tone contrast are shown at the time point of MMN peak amplitude on electrode Fz (Right). **(B)** Grand average traces recorded from one pair of electrodes on the left and right sides (Left) and grand average scalp topographic maps (Right) of MMN evoked by the pure-tone contrast in the control (Upper, *n* = 14), RTL (Central, *n* =11), and RNTL (Lower, *n* = 13) groups. Gray bars indicate the time window in which MMN amplitude was calculated.

Two sets of electrodes on the left (F3, FC1, FC5, C3) and right (F4, FC2, FC6, C4) sides of the scalp were identified as the regions of interest (Doeller et al., 2003; Luo et al., 2006). The amplitudes of MMN recorded at the four electrodes on each side were averaged within a time window from 20 ms before the peak of MMN between 100 and 300 ms recorded from electrode Fz to 20 ms after that peak (as indicated by the gray bars in the left panels of Figures 2A,B) (Wang et al., 2013). The MMN amplitude would be set to zero for all subsequent analyses when the averaged value was positive (Robson et al., 2014). The MMN latency was measured between 100 and 300 ms at electrode Fz. Then, the lateralization index (LI) for each stimulus condition and each participant was calculated by using the MMN amplitudes of the left and right sides. The LI was calculated by the following formula:


$$\begin{array}{l} \text{LI }=\;(\text{left MMN amplitude }-\text{ right MMN amplitude})\text{/}\\ \;(\text{left MMN amplitude + right MMN amplitude}). \end{array}$$

An index value of −1 indicates a lateralized response entirely in the right hemisphere and an index value of +1 indicates a lateralized response entirely in the left (Seghier, 2008). Supplementary Figure 1 shows the relationship between the LI for each stimulus condition (lexical-tone contrast and pure-tone contrast) and the time post-stroke onset in the RTL and RNTL groups. The findings showed no significant relationship.

A Shapiro–Wilk test was performed to check the normality of Token test scores, bilateral MMN amplitudes, and MMN latencies and LI values for each group. The results showed that Token test scores and MMN latencies were normally distributed in all groups, but bilateral MMN amplitudes and LI values were not normally distributed in some groups and stimulus conditions. Welch ANOVA was performed to test the significance of differences in Token test scores among groups, given that the Token test scores among groups (control, RTL, and RNTL) failed the assumption for homogeneity of variance tested using Levene's test. *Post-hoc* pairwise comparisons with the Games–Howell test were performed. For assessing possible lateralization effects, we performed a Wilcoxon signed-rank test between bilateral MMN amplitudes for each stimulus condition and each group. Moreover, the LI for each stimulus condition and each group were assessed by using a one-sample Wilcoxon signed-rank test and compared with the value 0. An LI index value significantly <0 indicates rightward lateralization and a value significantly >0 indicates leftward lateralization. We performed the Kruskal–Wallis *H*-test and used Dunn's test as a *post-hoc* test to determine whether the LI and unilateral MMN amplitudes showed significant differences among groups (control, RTL, RNTL) for each stimulus condition. For exploring the relationships among LIs obtained under different stimulus conditions, lesion volume, and speech-comprehension ability, we obtained the Spearman's correlation coefficients for the following correlations: (i) the correlations between lesion volume and Token test scores in the RTL and RNTL groups; (ii) the correlations between lesion volume and LI for each stimulus condition in the RTL and RNTL groups; (iii) the correlations between the Token test scores and LI for each stimulus condition and each group. The statistical difference was considered significant with the alpha-level set as *P* < 0.05 for all tests. One-way ANOVA was performed to test whether MMN latency showed significant differences among groups, and *post-hoc* pairwise comparisons with the Bonferroni test were performed. All data are expressed as mean ± SE or median ± minimum/maximum value. The correlation coefficients were marked as *r*_s_. SPSS V13 software (IBM, USA) and OriginPro V8 software (OriginLab Corp., USA) were used for statistical analysis and graph plotting.

## Results

### Speech comprehension ability for each group and its correlation with lesion volume

There was a significant difference in Token test scores among groups as determined by Welch ANOVA [*F*_(2, 19.50)_ = 6.31, *P* < 0.01]. *Post-hoc* pairwise comparisons with Games–Howell's test showed that Token test scores in the RTL group were lower than those in the control group (95% confidence interval= −6.17, −0.93; *P* < 0.01) and those in the RNTL group (95% confidence interval= −6.05, −0.66; *P* < 0.05; Figure 1C, Left). Moreover, a significant negative correlation was found between lesion volume and Token test scores in the RTL group (*r*_s_ = −0.84, *P* < 0.01; Figure 1C, Central); that is, the larger lesion volume, the worse speech comprehension ability of Mandarin Chinese. And no significant correlation was found between lesion volume and Token test scores in the RNTL group (*r*_s_ = 0.27, *P* > 0.05; Figure 1C, Right).

### MMN and LI for the left and right sides of the scalp in lexical and pure tone conditions

MMN waveforms were prominent in both lexical and pure tone conditions as illustrated by sample traces of grand average MMN in response to the lexical-tone contrast and to the pure-tone contrast (Figures 2A,B, left panels). The MMN in the control group under each stimulus condition and that in the RNTL group under the pure-tone contrast were stronger in magnitude when recorded on the right side of the scalp than those recorded on the left side. In contrast, MMN of the RTL group in response to the lexical-tone contrast demonstrated a swapped pattern: it was stronger in magnitude when recorded on the left side of the scalp than that recorded on the right side. More detailed latencies of the MMN between groups under each condition showed no significant difference across groups (Supplementary Figure 2). The right panels of Figures 2A,B show the scalp topographic maps constructed with grand average MMN in response to the lexical-tone contrast and the pure-tone contrast for the control (*Upper*), RTL (*Central*), and RNTL (*Lower*) groups. The MMN topographic maps were obviously lateralized in strength to the right side of the scalp in the control and RNTL groups under each stimulus condition, whereas they were obviously lateralized in strength to the left side of the scalp in the RTL group.

**Figure 3:**
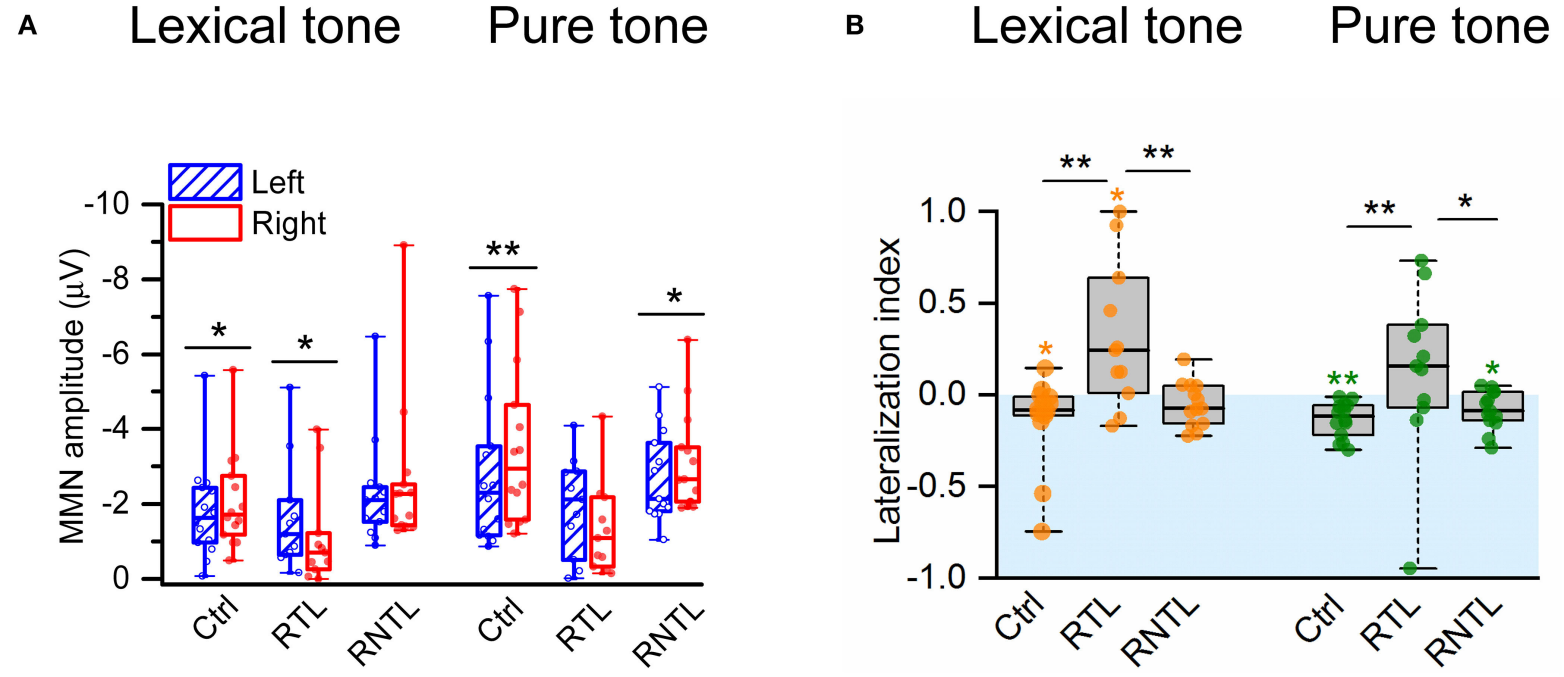
MMN amplitudes and lateralization index (LI) were recorded from four electrodes on the left (F3, FC1, FC5, C3) and four electrodes on the right (F4, FC2, FC6, C4) sides of the scalp. **(A)** MMN was significantly larger in amplitude on the right side of the scalp than on the left in the control group for the lexical-tone contrast and the pure-tone contrast and in the RNTL group for the pure-tone contrast but larger in amplitude on the left side of the scalp than on the right in the RTL group for the lexical-tone contrast. **(B)** The comparisons for LI within each group and among groups. The analysis within the group indicates that the LI was significantly less than zero (indicates right hemisphere lateralized response) in the control group for the lexical-tone contrast and the pure-tone contrast and in the RNTL group for the pure-tone contrast but greater than zero (indicates left hemisphere lateralized response) in the RTL group for the lexical-tone contrast. The analysis among groups indicates that the LI in the RTL group was larger than that in the control group and the RNTL group for each stimulus condition. Box plots depict medians with interquartile ranges and whiskers represent the minimum and maximum values. *P < 0.05, **P < 0.01.

The analysis of MMN amplitudes calculated from four pairs of electrodes on the left (F3, FC1, FC5, C3) and right (F4, FC2, FC6, C4) sides of the scalp in the individual participant (Figure 3A) demonstrates the swapped hemispheric lateralization of the MMN responses to the lexical-tone contrast in the RTL group. Wilcoxon signed-rank test showed that the MMN response was significantly lateralized to the right side of the scalp in the control group for both conditions (lexical tone, *Z* = −2.48, *P* < 0.05; pure tone, *Z* = −3.30, *P* < 0.01) and in the RNTL group for the pure-tone contrast (*Z* = −2.20, *P* < 0.05), whereas the MMN response was significantly lateralized to the left side of the scalp in the RTL group for the lexical-tone contrast (*Z* = −2.13, *P* < 0.05; Figure 3A). No other significant results between bilateral MMNs were shown in the RTL or RNTL group.

The swapped pattern of hemisphere lateralization of MMN responses to the lexical-tone contrast was also revealed by the LI. A one-sample Wilcoxon signed-rank test showed that the LI was significantly less than zero (i.e., lateralized to the right hemisphere) in the control group under each stimulus condition (lexical tone: median value = −0.08, *P* < 0.05; pure tone: median value = −0.12, *P* < 0.01) and in the RNTL patients for the pure-tone contrast (median value = −0.09, *P* < 0.05), whereas the LI was significantly greater than zero (i.e., lateralized to the left hemisphere) in the RTL group under the lexical-tone contrast (median value = 0.24, *P* < 0.05; Figure 3B). The Kruskal–Wallis H test showed significant differences in LI among groups for both conditions (lexical tone: $X_{\left(2\right)}^{2}$ = 9.67, *P* < 0.01, with a mean rank LI score of 15.00 for the control group, 28.18 for the RTL group, and 17.00 for the RNTL group; pure tone: $X_{\left(2\right)}^{2}$ = 9.20, *P* < 0.05, with a mean rank LI score of 14.07 for the control group, 27.55 for the RTL group, and 18.54 for the RNTL group). *Post-hoc* pairwise comparisons with Dunn's test showed that LIs for both stimulus conditions in the RTL group were larger than those in the control (lexical tone: *P* < 0.01; pure tone: *P* < 0.01) and the RNTL groups (lexical tone: *P* < 0.01; pure tone: *P* < 0.05; Figure 3B). No significant LI difference was observed in either stimulus condition between the control and the RNTL groups.

To explore the causes of the swapped hemisphere dominance, we analyzed the differences in unilateral MMN amplitudes across groups. The Kruskal–Wallis *H*-test showed that the right MMN amplitudes across groups were significantly different for each stimulus condition (lexical tone: $X_{\left(2\right)}^{2}$ = 9.42, *P* < 0.01, with a mean rank MMN amplitude score of 17.29 for the control group, 28.00 for the RTL group, and 14.69 for the RNTL group; pure tone: $X_{\left(2\right)}^{2}$ = 11.76, *P* < 0.01, with a mean rank MMN amplitude score of 15.71 for the control group, 29.18 for the RTL group, and 15.38 for the RNTL group). *Post-hoc* pairwise comparisons with Dunn's test revealed that the MMN amplitude on the right scalp for each stimulus condition in the RTL group was lower than that in the control (lexical tone: *P* < 0.05; pure tone: *P* < 0.01) and RNTL groups (lexical tone: *P* < 0.01; pure tone: *P* < 0.01). However, MMN amplitudes on the right scalp between the control and RNTL groups were significantly different for neither the lexical-tone contrast (*P* > 0.05) nor the pure-tone contrast (*P* > 0.05). Moreover, MMN amplitudes on the left scalp among groups were significantly different for neither the lexical-tone contrast ($X_{\left(2\right)}^{2}$ = 3.20, *P* > 0.05) nor the pure-tone contrast ($X_{\left(2\right)}^{2}$ = 1.74, *P* > 0.05). The reduction in the MMN amplitude in the right hemisphere of the RTL group indicates a swapping of brain dominance.

### Correlations between lesion volume and LI and those between LI and token test scores

**Figure 4:**
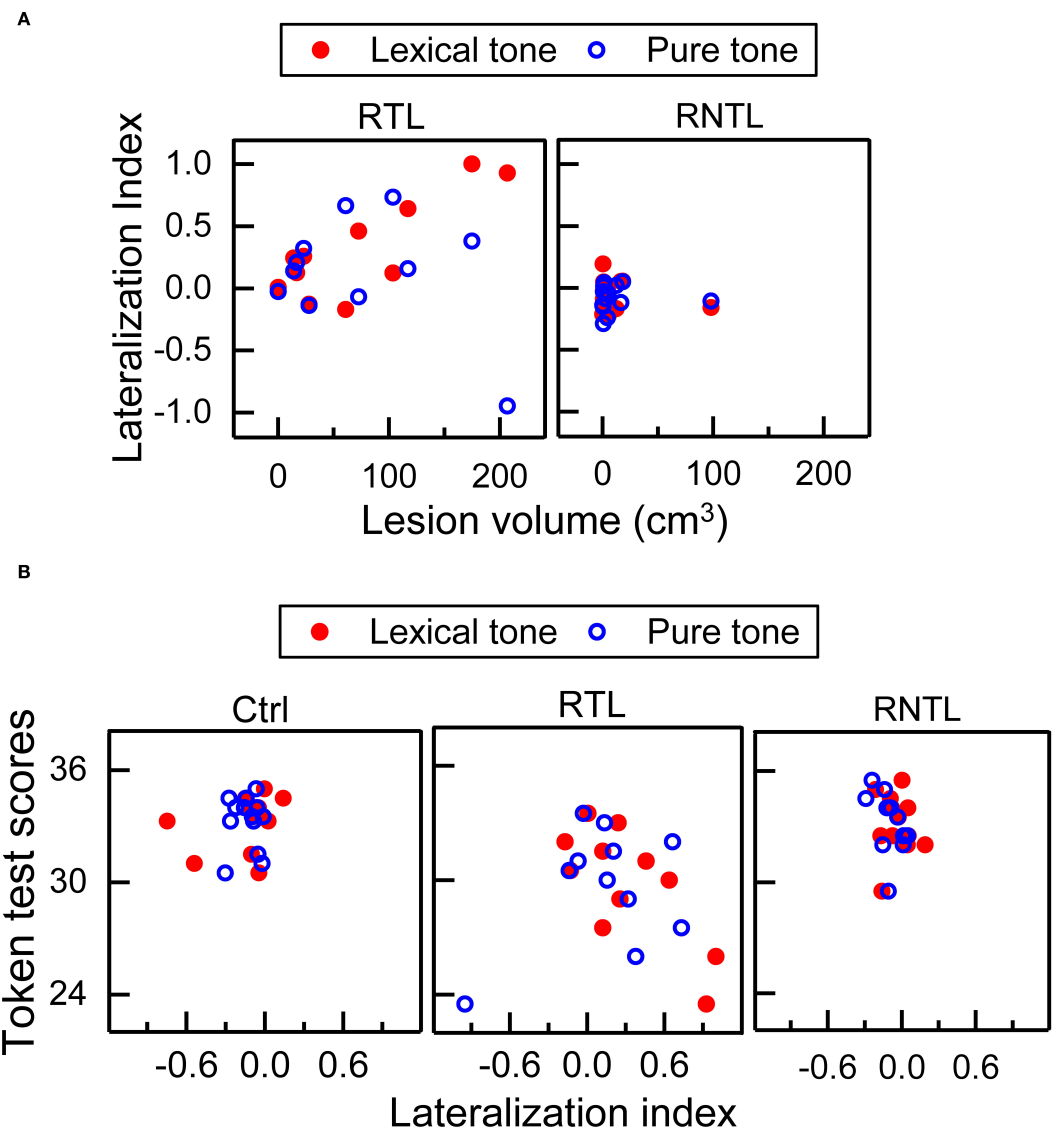
Correlations between lesion volume and lateralization index (LI), and those between LI and Token test scores. **(A)** Significant correlation between lesion volume and LI was only found in the RTL group for the lexical-tone contrast—more lesion volume corresponding to less right hemisphere involvement. **(B)** Significant correlation between LI and Token test scores was only found in the RTL group for the lexical-tone contrast—less right hemisphere involvement corresponding to worse speech comprehension ability of Mandarin Chinese.

Our findings showed significant correlations between lesion volume and LI, and between LI and Token test scores in the RTL group. Significant correlation between lesion volume and LI was only found in the RTL group for the lexical-tone contrast (*r*_s_ = 0.72, *P* < 0.05; Figure 4A, Left), indicating that a larger lesion volume corresponds to less right hemisphere involvement. Significant correlation was also observed between LI and Token test scores in the RTL group for the lexical-tone contrast (*r*_s_ = −0.74, *P* < 0.01; Figure 4B, Central), suggesting that less right hemisphere involvement corresponds to impaired speech comprehension ability for Mandarin Chinese. No other significant correlations were observed among lesion volume or Token test scores (Figure 4). The detailed results of correlation analysis between the LI for each stimulus condition (lexical-tone contrast and pure-tone contrast) and lesion volume/Token test scores are shown in Supplementary Table 2.

## Discussion

In the present study, we investigated how brain damage in stroke patients affects the labor division of the brain hemispheres for auditory processing of linguistic tones. Our study demonstrates that RTL injury results in swapped dominance of brain hemispheres in the preattentive auditory processing of Chinese lexical tones, suggesting that the RTL is a core area for early-stage auditory tonal processing.

Since the early auditory processing of lexical tones was lateralized to the right hemisphere for the control group and the left hemisphere for the RTL group (Figures 2, 3), the findings demonstrate swapped dominance of brain hemispheres in preattentive auditory processing of Chinese lexical tones after RTL stroke. Meta-analyses of lexical-tone processing have suggested significant activations in both temporal lobes in response to lexical tones (Kwok et al., 2017; Liang and Du, 2018), but non-tonal language studies only showed significant activations in the left temporal lobe (Kwok et al., 2017). Lexical-tone studies demonstrate more activations in the RTL than in the left (Liang and Du, 2018). Some researchers suggest that the appearance of language impairments in right-handed patients with right brain injury represents atypical language lateralization before stroke (Gajardo-Vidal et al., 2018). Therefore, the right lateralization in the control group and the impairment of right lateralization in the RTL group would not have been caused by atypical language lateralization. Moreover, the comparisons of LI for the lexical tone and the pure tone within and among groups showed that the RTL group exhibited left lateralization while the control and RNTL groups exhibited right lateralization at a preattentive stage (Figure 3B). Our findings support our hypothesis that RTL injury changes the right hemisphere dominance during the early auditory processing of lexical tones. Notably, both lexical and pure tones reflecting varied spectral information are dominantly processed in the right hemisphere (Zatorre and Belin, 2001; Schonwiesner et al., 2005), which is consistent with the acoustic hypothesis that the hemispheric specialization for processing auditory perception depends on the acoustic structure of the auditory input (Zatorre and Belin, 2001; Schonwiesner et al., 2005). Our results demonstrate that only RTL injury swaps the hemisphere dominance in early auditory processing of lexical tone that carries semantic information. In the RTL group, the reduced right hemisphere involvement for processing lexical tones at a preattentive stage corresponds to the worse speech-comprehension ability for Mandarin Chinese (Figure 4B, Central). Although left hemisphere dominance for processing language has been demonstrated since the 1870s (Broca, 1861; Wernicke, 1874; Tyler et al., 2010, 2011; Teki et al., 2013), the present study suggests that language disorders can occur after injury in the right hemisphere, especially in the RTL.

Although the RTL group exhibited left hemispheric lateralization for processing lexical tones at an early stage, this does not necessarily mean that the left hemisphere compensates for the impaired auditory function of the right hemisphere. The worse speech-comprehension ability for Mandarin Chinese in the RTL group was associated with a reduced right hemisphere-lateralized response for early auditory processing of lexical tones (Figure 4B, Central). This is consistent with a previous study in which the RTL was shown to correlate with speech comprehension (Walenski et al., 2019). Moreover, we found a significant association between lesion volume and LI for the lexical-tone contrast at a preattentive stage in the RTL group: the larger the lesion volume, the lower the right hemisphere-lateralized response (Figure 4A, Left). Similar associations between larger lesion volumes and lower functional improvement have been also found in rats after stroke (Sasaki et al., 2016). Since the RTL group mainly showed lesions in the RTL (Figure 1A), we think that the disappearance of right hemisphere dominance may be caused by the decreased neural activity involved in lexical-tone perception in the right hemisphere. We further suggest that RTL injury impairs the speech comprehension of tonal languages. A previous study demonstrated that the speech comprehension of non-tonal languages can be impaired by right brain damage and that the most frequently impaired language task is auditory sentence-to-picture matching (Gajardo-Vidal et al., 2018).

Notably, in RTL stroke patients, the neural activity revealed by the MMN amplitude on the right scalp was significantly lower than those in the control and RNTL groups, but the neural activity of the left hemisphere in the RTL group was not significantly different. This is in line with the findings of previous studies, which showed that patients with unilateral brain damage may show diminished response on the injured side (Alho et al., 1994; Deouell et al., 2000; Tyler et al., 2011). The swapped hemisphere dominance in early auditory processing of lexical tone in patients with RTL stroke is obviously caused by the decreased MMN amplitude of the right hemisphere. The previous studies indicated that the source generator of MMN originates from the left and right auditory regions (Naatanen et al., 1997; Kujala et al., 2002). Naatanen et al. (1997) demonstrated the source generators of MMN in the left and right auditory cortices for speech and non-speech sounds in their well-known MEG study. If the lesion is in the RTL, then the MMN activity on the right side would be reduced. The more extensive the damage is, the greater the reduction in MMN on the right side. The MMN amplitudes of the left hemisphere (uninjured side) showed no significant difference across groups (Figure 3A). This is inconsistent with the findings of previous studies in which decreased neural activity on the injured side was suggested to result in increased neural activity on the uninjured side in speech perception (Becker and Reinvang, 2007; Tyler et al., 2010, 2011; Teki et al., 2013). Similar findings have been reported in another study showing enhanced interactions between the hemispheres in patients with RTL epilepsy but not in control subjects or patients with RNTL epilepsy (He et al., 2018). Some of the possible reasons to explain why the left MMN amplitude in the RTL group was not increased can be summarized as follows: (i) patients in the RTL group were mostly in the acute stage (post-stroke onset <3 months, Supplementary Table 1), and stable compensation of the left hemisphere for lexical-tone perception may not yet have occurred; (ii) the spectral variation of lexical tones is a basal variation in acoustic patterns, which might be difficult for the left hemisphere to compensate for; and (iii) the lesion volume and the lesion area in RTL stroke patients varies substantially, and it might be difficult to form a stable pattern. Moreover, the decreased MMN amplitude of the right side and unchanged MMN amplitude of the left side in the RTL group may result in the patients not able to discriminate the lexical tones as well as the controls. Future research should add behavioral experiments to explore whether the RTL group has impairments in both MMN amplitude in lexical-tone contrast and the ability to distinguish the lexical tones.

Our present study shows that the RNTL injury also affects the hemisphere dominance in response to the lexical-tone contrast (Figure 3). The RNTL may be the facultative brain regions in early auditory processing of lexical tone, and the RTL may be the obligatory brain region. In addition to the RTL, other brain areas of the right hemisphere, such as the right white matter (Zhao et al., 2016), the right basal ganglia (Chang and Kuo, 2016), and the right visual cortex (Kwok et al., 2015) may also participate in lexical-tone perception. Patients with RNTL injuries mainly showed lesions in the right periventricular white matter, the right basal ganglia, and the right occipital lobe (Supplementary Table 1 and Figure 1B). These results may indicate the importance of the cooperation and connectivity of the multiple brain areas in the early auditory processing of lexical tones, and a special pattern might form when a specific brain region is damaged. Unlike the lexical-tone contrast, the RNTL group still showed right hemisphere dominance in response to the pure-tone contrast (Figures 2, 3). This is consistent with the results of previous studies reporting that the processing of spectral variation for non-speech stimuli mainly occurs in the RTL (Opitz et al., 2002; Schonwiesner et al., 2005). Therefore, early auditory processing of pure tones was not impaired in the RNTL group.

Several limitations should be noted when interpreting our findings. First, the sample size was relatively small. We spent 2 years and recruited 30 stroke patients and 14 healthy control participants. Among these patients, we excluded five stroke patients with severe hearing loss, and one patient withdrew consent. Therefore, the stroke patients were well-characterized. Second, although the difference in age across groups was not significant, participant age showed substantial variation, implying that the sample may not be representative of younger stroke sufferers. Third, since lexical tones and music have the same patterns (Nan and Friederici, 2013; Chen et al., 2018), detailed information about the musical experience of the participants should have been collected. Nevertheless, since the aim of our study was to investigate the effect of right brain injury on the hemispheric dominance of lexical tones, we did not consider the effects of musical training when recruiting subjects. Stroke patients were not always ready for recruitment, and their availability would have been reduced even further if the musical training-related factor had been applied. Fourth, our study lacked exact control between the two stimulus conditions. Because the recorded MMN response in the pure-tone contrast occurred ~150 ms after the onset of the stimuli under a passive auditory oddball paradigm (Aaltonen et al., 1993), we selected a 200-ms pure tone and a 500-ms inter-stimulus onset interval (ISI) for the pure-tone contrast (Wang et al., 2021). Notably, the MEG data for the neural basis of perceptual processing of lexical tones indicated a left hemispheric dominance for detecting large lexical-tone changes and small deviant contrasts involving less left hemispheric activation in the auditory cortex and greater activation in the right frontal cortex at a later time window (Hsu et al., 2014). The cross-category contrasts also revealed larger MMN responses than within-category contrasts in the left scalp, but not in the right scalp (Xi et al., 2010; Zhang et al., 2011). In addition, an MMN study investigating the effect of allophonic variation on the mental representation and neural processing of lexical tones suggested that activation of the allophonic tonal variants can lead to right-hemisphere-dominant processing of lexical tones, which are otherwise categorically processed via recruitment of both left and right hemispheres (Li and Chen, 2015).

We assessed the speech-comprehension ability for Mandarin Chinese (a tonal language) by the Token test, a language task involving auditory sentence-to-picture matching. The performance in the speech-comprehension task in the RTL group was worse than that in the RNTL and control groups (Figure 1C, Left). In the RTL group, the speech-comprehension ability for Mandarin Chinese negatively correlated with the lesion volume (Figure 1C, Central), indicating a causal role of the RTL in Mandarin speech perception. Considering the growing awareness that aphasia following a stroke can include deficits in other cognitive functions (Schumacher et al., 2019) and the importance of accurately representing lexical-tone information for hearing-impaired Mandarin speakers (Li et al., 2019; Chen et al., 2020), our study highlights the necessity of rehabilitating the language functions of tonal language speakers who suffer from RTL injury and applying formal lexical-tone-related communication tests in clinical assessment and rehabilitation for patients who are speakers of tonal languages and experience brain injury and communication disorders.

To summarize, our findings showed swapped dominance of lateralization from the right to the left hemisphere in patients with RTL injuries but not in those with RNTL injuries, indicating that the RTL is a core area for auditory tonal processing at an early stage or a preattentive stage. These findings indicate the necessity of rehabilitating language functions of tonal language speakers who experience RTL injury.

## Data availability statement

The original contributions presented in the study are included in the article/Supplementary material, further inquiries can be directed to the corresponding authors.

## Ethics statement

The studies involving human participants were reviewed and approved by Ethics Committee of the First Affiliated Hospital, University of Science and Technology of China. The patients/participants provided their written informed consent to participate in this study.

## Author contributions

LC designed the research. YW and MW performed the research. YW, XL, XG, XW, YQ, and RA analyzed data. LC, XF, HL, BQ, MW, RQ, YW, and XL wrote the paper. All authors contributed to the article and approved the submitted version.

## Funding

This work was supported by the National Natural Science Foundation of China (Grants 81970886, 81570915, and 81870723) and Anhui Provincial Natural Science Foundation (Grant 30973084).

## Conflict of interest

The authors declare that the research was conducted in the absence of any commercial or financial relationships that could be construed as a potential conflict of interest.

## Publisher's note

All claims expressed in this article are solely those of the authors and do not necessarily represent those of their affiliated organizations, or those of the publisher, the editors and the reviewers. Any product that may be evaluated in this article, or claim that may be made by its manufacturer, is not guaranteed or endorsed by the publisher.
