# Supplementary material for: Brain hemispheres with right temporal lobe damage swap dominance in early auditory processing of lexical tones
Source: Front Neurosci. 2022 Aug 26;16:909796. doi: 10.3389/fnins.2022.909796 (PMC9459135; doi:10.3389/fnins.2022.909796)
Supplement: Supplementary file 1 [file Table_1.doc]

**Supplementary Table 1. Demographic characteristics and clinical findings of patients with right brain damage (n = 24) and control subjects (n = 14).**

|  | Subject | Sex | Age | MPO | Left ear* | Right ear* | Token test | Lesion type | Lesion volume† | Lesion areas |
| --- | --- | --- | --- | --- | --- | --- | --- | --- | --- | --- |
| RTL | 1 | M | 58 | 0.3 | 20 | 35 | 33 | I | 14.1 | BG, Insular, T |
|  | 2 | M | 61 | 1.4 | 28.75 | 36.25 | 31 | I | 72.8 | T, O, P |
|  | 3 | M | 66 | 1.2 | 16.25 | 13.75 | 33.5 | I | 0.3 | T |
|  | 4 | M | 42 | 0.2 | 27.5 | 25 | 30.5 | I | 28.1 | T, Insular, P, CSO |
|  | 5 | M | 37 | 9 | 26.25 | 13.75 | 32 | I | 61.0 | T, BG, Insular |
|  | 6 | M | 62 | 1.5 | 22.5 | 36.25 | 27.5 | I | 103.6 | T, O, P, F |
|  | 7 | M | 75 | 0.2 | 45 | 45 | 31.5 | I | 16.9 | BG, PVWM, P, T, F |
|  | 8 | M | 45 | 0.4 | ‡ | ‡ | 29 | I | 23.3 | Insular, T, F, P |
|  | 9 | F | 76 | 2.5 | 55 | 51.25 | 23.5 | I | 207.0 | P, F, T, O |
|  | 10 | F | 56 | 2.2 | 53.75 | 45 | 30 | H | 117.3 | T, F, P |
|  | 11 | M | 51 | 0.3 | 36.25 | 17.5 | 26 | I | 174.8 | T, P, F, Insular |
| RNTL | 12 | F | 49 | 1.3 | 22. 5 | 20 | 34 | I | 2.8 | PVWM, BG |
|  | 13 | M | 63 | 3.1 | 43.75 | 42.5 | 32.5 | H | 18.2 | BG |
|  | 14 | F | 63 | 12 | ‡ | ‡ | ‡ | H | 5.2 | Th, PVWM, Brainstem |
|  | 15 | M | 53 | 0.2 | 40 | 27.5 | 32.5 | I | 1.5 | PVWM |
|  | 16 | M | 53 | 0.2 | 17.5 | 17.5 | 32 | I | 0.4 | PVWM |
|  | 17 | M | 37 | 24.9 | 21.25 | 17.5 | 34.5 | I | 0.8 | BG, PVWM |
|  | 18 | M | 63 | 1.2 | 40 | 37.5 | 35 | I | 0.3 | PVWM, Brainstem |
|  | 19 | M | 61 | 0.6 | 22.5 | 18.75 | 33.5 | I | 0.9 | PVWM, BG |
|  | 20 | M | 43 | 36 | 18.75 | 13.75 | 32 | I | 1.1 | PVWM, BG |
|  | 21 | M | 56 | 0.1 | 46.25 | 46.25 | 32.5 | I | 12.5 | O |
|  | 22 | M | 48 | 0.4 | 40 | 26.25 | 34 | I | 16.6 | O, Th |
|  | 23 | M | 58 | 1.6 | 31.25 | 33.75 | 29.5 | I | 98.2 | O, P |
|  | 24 | M | 44 | 0.7 | 36.25 | 27.5 | 35.5 | I | 4.6 | BG |
| Control | 25 | F | 46 | § | 18.75 | 13.75 | 34.0 | § | § | § |
|  | 26 | M | 62 | § | 25 | 23.75 | 34.5 | § | § | § |
|  | 27 | M | 49 | § | 26.25 | 22.5 | 34.5 | § | § | § |
|  | 28 | M | 63 | § | 22.5 | 20 | 33.0 | § | § | § |
|  | 29 | F | 48 | § | 17.5 | 22.5 | 35.0 | § | § | § |
|  | 30 | F | 48 | § | 10 | 16.25 | 31.0 | § | § | § |
|  | 31 | M | 57 | § | 42.5 | 30 | 34.0 | § | § | § |
|  | 32 | M | 64 | § | 12.5 | 12.5 | 33.0 | § | § | § |
|  | 33 | M | 48 | § | 18.75 | 18.75 | 31.5 | § | § | § |
|  | 34 | M | 46 | § | 23.75 | 22.5 | 34.0 | § | § | § |
|  | 35 | F | 44 | § | 18.75 | 18.75 | 30.5 | § | § | § |
|  | 36 | F | 48 | § | 15 | 21.25 | 33.5 | § | § | § |
|  | 37 | M | 63 | § | 43.75 | 36.25 | 34.0 | § | § | § |
|  | 38 | M | 42 | § | 28.75 | 26.25 | 33.5 | § | § | § |
|  | χ2/F | 1.80 | 0.97 | - | 6.31 | 5.08 | 3.24 | - | - | - |
|  | *P* | 0.41 | 0.61 | - | 0.008 | 0.08 | 0.20 | - | - | - |

RTL = right temporal lobe; RNTL = right non-temporal lobe. F (in the “Sex” column) = female; M = male; MPO = months post stroke onset; H = hemorrhagic; I = ischemic; BG = basal ganglia; F (in the “Lesion areas” column) = frontal; O = occipital; P = parietal; PVWM = peri ventricular white matter; T = temporal. * indicates that the unit of measure for hearing thresholds is decibel/hearing level (dB/HL). † indicates that the unit of display for lesion volume is cm³. ‡ indicates that the hearing threshold data of number 8 in the RTL group and number 3 in the RNTL group and Token test scores of number 3 in the RNTL group were not collected, but the subjects or their family members reported no hearing problem before right brain damage. § indicates that the control subjects had no relevant data.

**Supplementary Table 2. The correlation results between lateralization index (LI) and lesion volume/Token test scores.**

|  | Lateralization index | | | | | | | | | | |
| --- | --- | --- | --- | --- | --- | --- | --- | --- | --- | --- | --- |
| Group | Lesion volume | | | | | | | Token test score | | | |
|  | Lexical tone | | | Pure tone | | | | Lexical tone | | Pure tone | |
|  | r_s_ | *P* | | r_s_ | | *P* | | r_s_ | *P* | r_s_ | *P* |
| Ctrl | - | | - | | - | | - | 0.26 | ns | -0.13 | ns |
| RTL | **0.61** | | **0.047** | | 0.05 | | ns | **-0.63** | **0.039** | -0.08 | ns |
| RNTL | 0 | | ns | | 0.39 | | ns | -0.23 | ns | -0.50 | ns |

The significant correlation results are marked by the bold face. r_s_ represents the correlation coefficient. ns = not significant.
