# Supplementary material for: Brain hemispheres with right temporal lobe damage swap dominance in early auditory processing of lexical tones
Source: Front Neurosci. 2022 Aug 26;16:909796. doi: 10.3389/fnins.2022.909796 (PMC9459135; doi:10.3389/fnins.2022.909796)
Supplement: Supplementary file 2 [file Image_1.pdf]

Supplementary Material

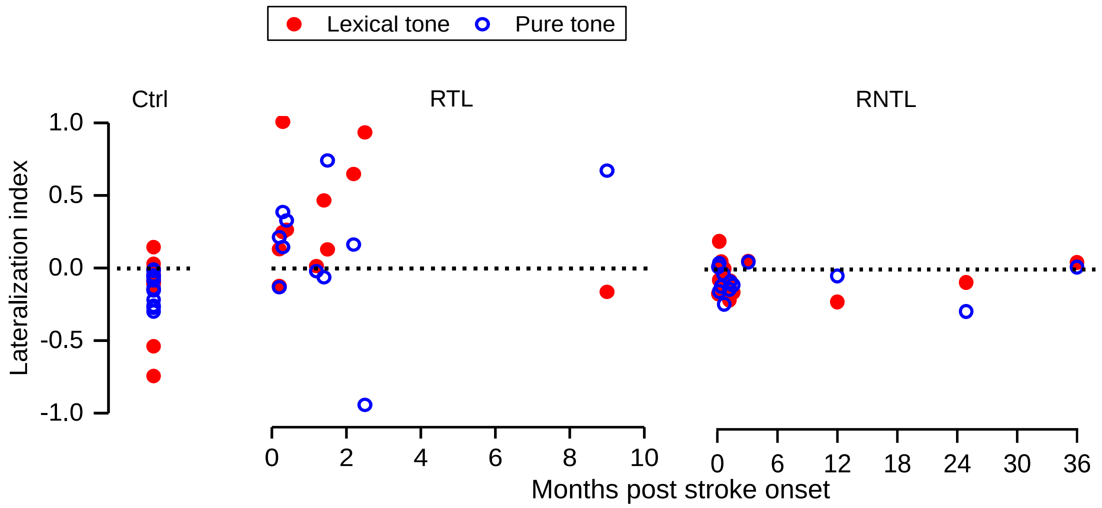

Supplementary Figure 1. Relationships between lateralization index (LI) and time post stroke onset. There was no significant relationship between LI for the lexical tone contrast or the pure tone contrast and time post stroke onset in the RTL or RNTL groups.

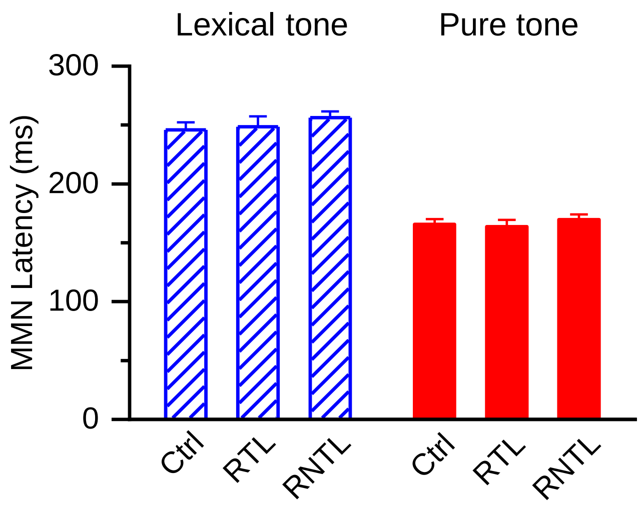

Supplementary Figure 2. MMN latency recorded from electrode Fz. There was no significant difference of MMN latencies between Ctrl, RTL and RNTL groups in lexical tone contrast or pure tone contrast.
